# Supplementary figures and images for: Simulations suggest walking with reduced propulsive force would not mitigate the energetic consequences of lower tendon stiffness
Source: PLoS One. 2023 Oct 26;18(10):e0293331. doi: 10.1371/journal.pone.0293331 (PMC10602298; doi:10.1371/journal.pone.0293331)

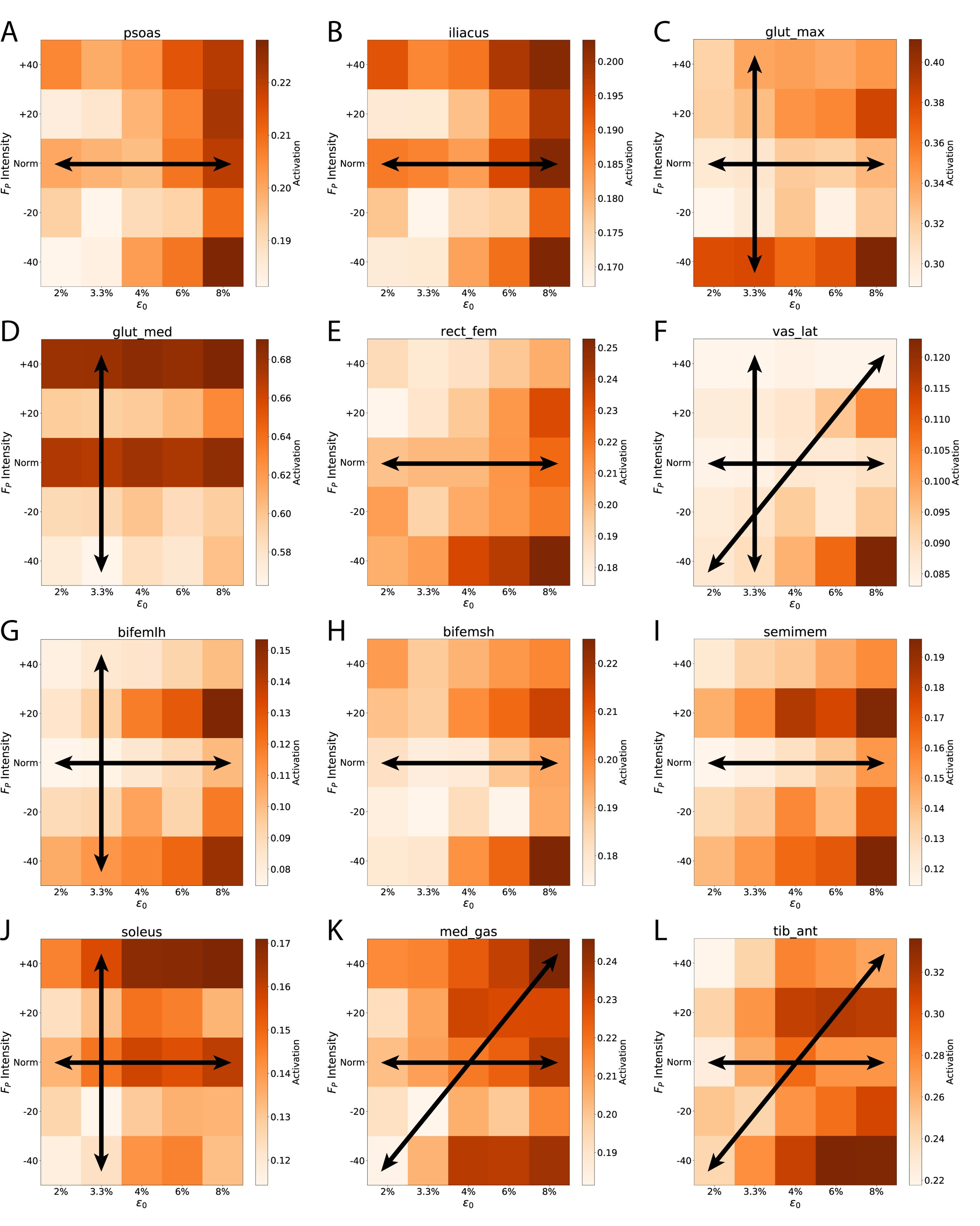

Supplement: S1 Fig — We show individual-muscle activation levels as a function of both kT and FP. ANOVA main effects are shown via arrows (horizontal, vertical, & diagonal) similar to Figs 2 & 4. Eleven of the 12 highest energy consuming muscles showed significant effects in activation level for kT (all but glut_med, panel D). Five of the 12 most costly muscles showed significant effects in activation level for FP. Three out of the 12 muscles displayed significant interactions between kT and FP for activation level. These data correspond with S1 Table. (TIF) [file pone.0293331.s001.tif]

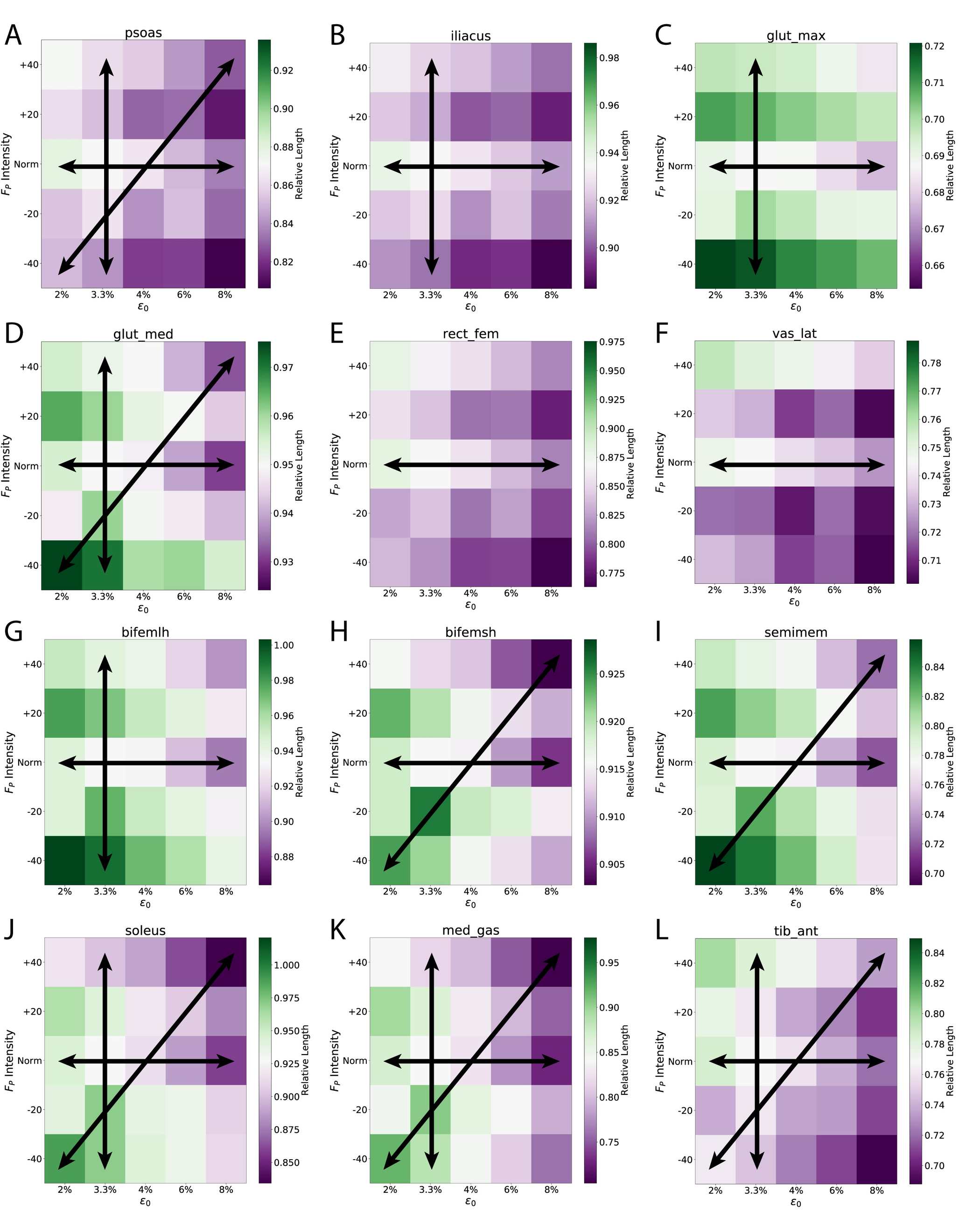

Supplement: S2 Fig — We show individual-muscle fiber lengths as a function of both kT and FP. The fiber lengths of all 12 of the costliest muscles were significantly impacted by kT. Additionally, 8 of these top 12 had significant effects for FP, while 7/12 showed significant interaction effects. These data correspond with S2 Table. (TIF) [file pone.0293331.s002.tif]

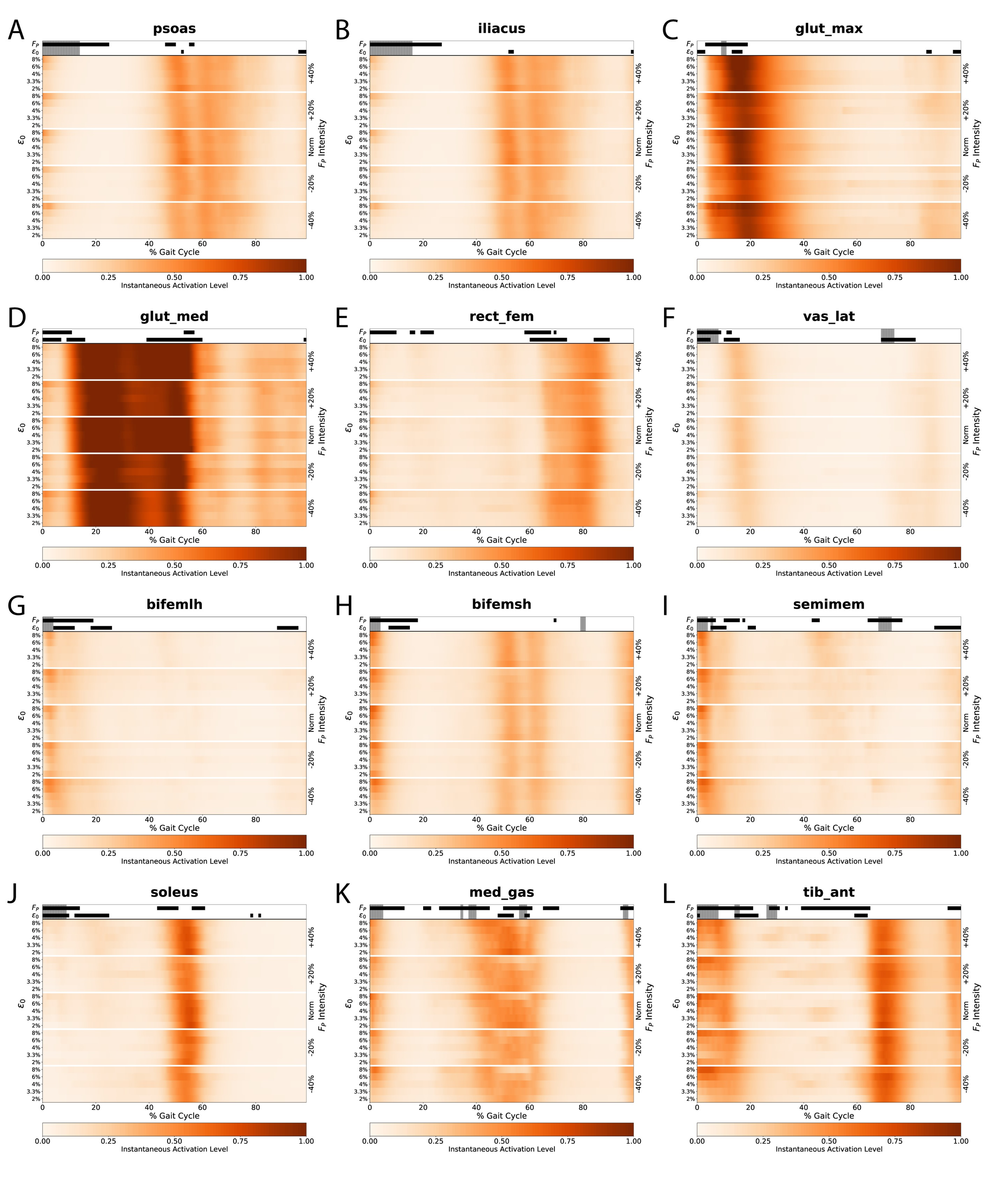

Supplement: S3 Fig — The instantaneous activation levels for highest consuming muscles highly aligned with the instantaneous metabolic costs (Fig 5). (TIF) [file pone.0293331.s003.tif]

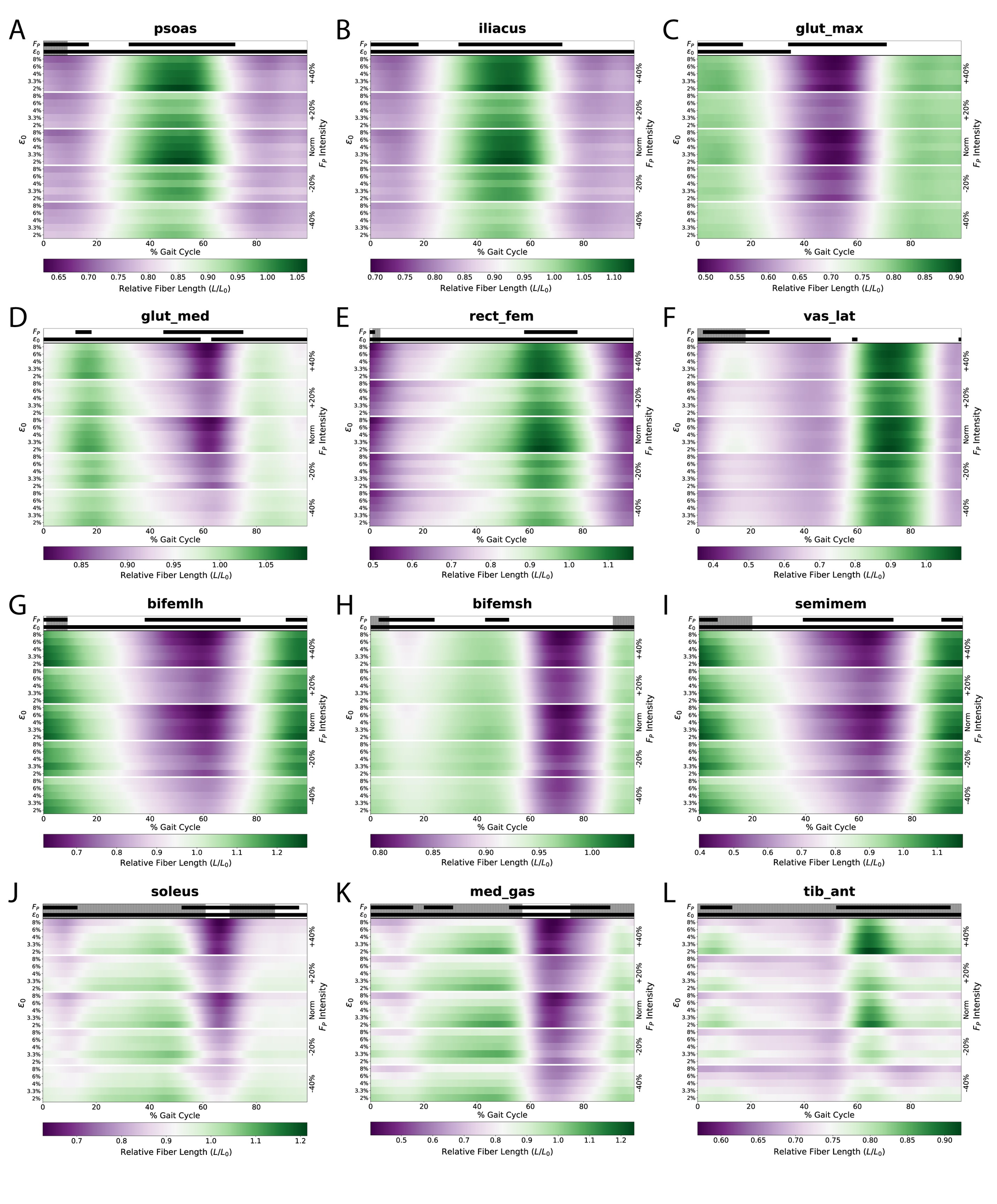

Supplement: S4 Fig — Viewing the instantaneous muscle fiber lengths, we see changes across the experimental conditions for both kT and FP. These showcase underlying changes in muscle actions as a result from the altered kT and FP. Large effects occur in the distal musculature, particularly for the solues, med_gas, and tib_ant (J, K, & L). (TIF) [file pone.0293331.s004.tif]

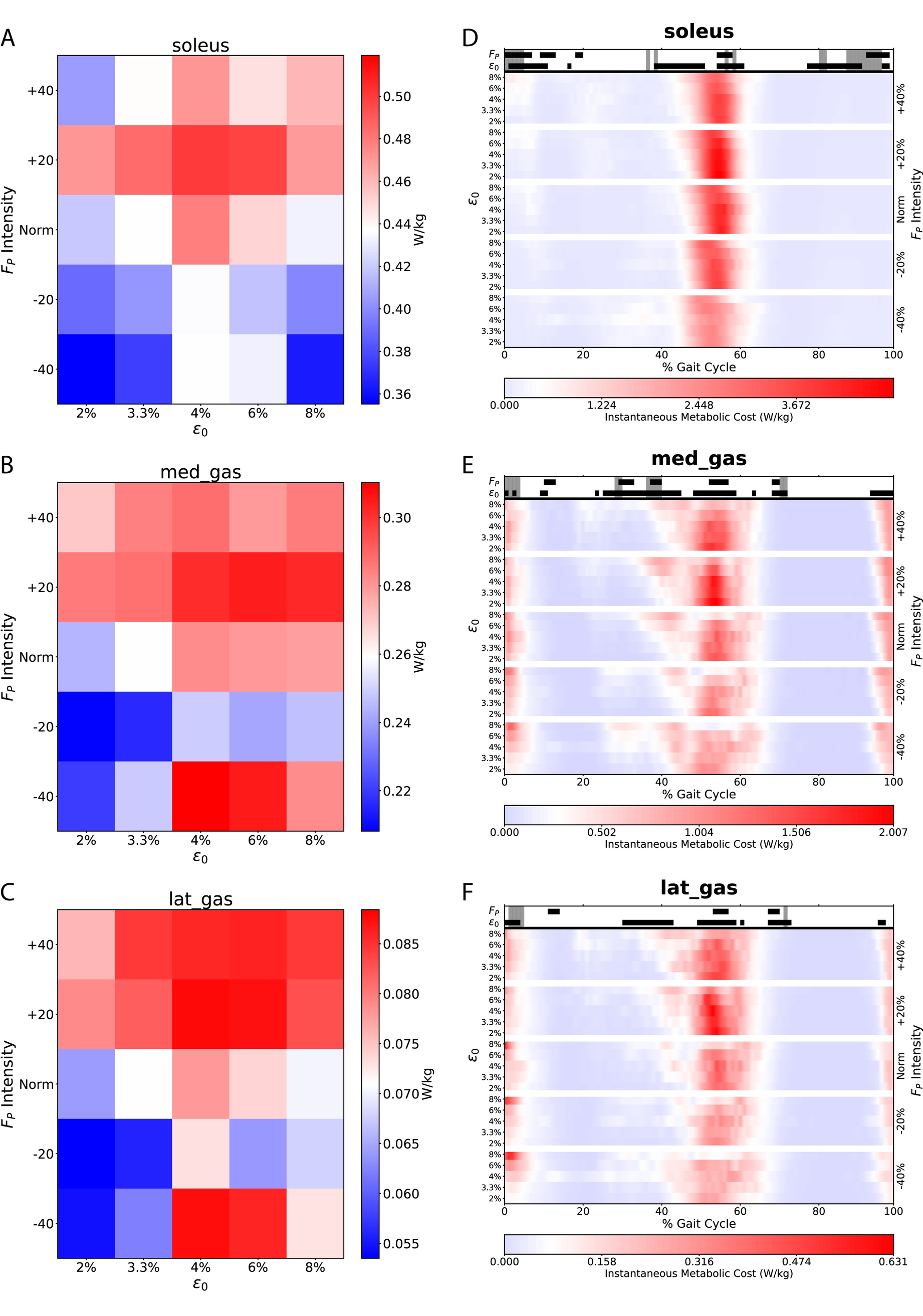

Supplement: S5 Fig — Due to their large influence on ankle moment and thus FP, we highlight the triceps surae metabolic cost on average (A-C) and across the gait cycle (D-F). One can see a high similarity between med_gas and lat_gas across FP and kT experimental conditions. This view of all three triceps surae muscles is inaccessible in other figures due to the small relative metabolic cost of lat_gas. (TIF) [file pone.0293331.s005.tif]

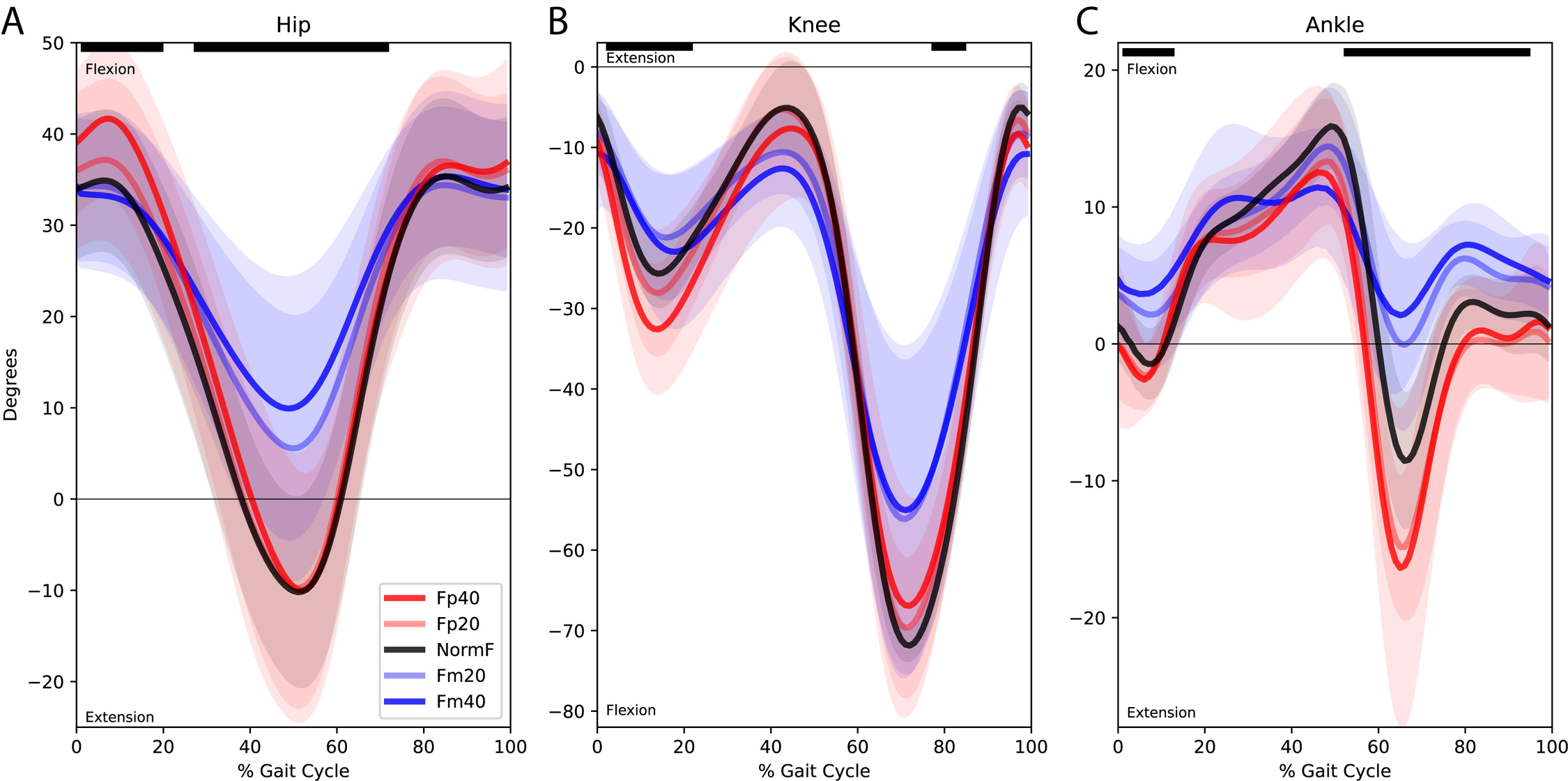

Supplement: S6 Fig — We show the influence of the FP biofeedback conditions on sagittal plane kinematics for the hip (A), knee (B), and ankle (C). Subject-averaged curves are surrounded by shading of ±1 standard deviation. We also calculated one-way repeated measures analysis of variance on the influence of FP condition on joint angles across the gait cycle using SPM. Like our other figures, a black bar at the top of each panel denotes a significant main effect for that instance of the gait cycle (1% increments). In general, reducing FP resulted in smaller dynamic ranges across all lower body joints. (TIF) [file pone.0293331.s006.tif]

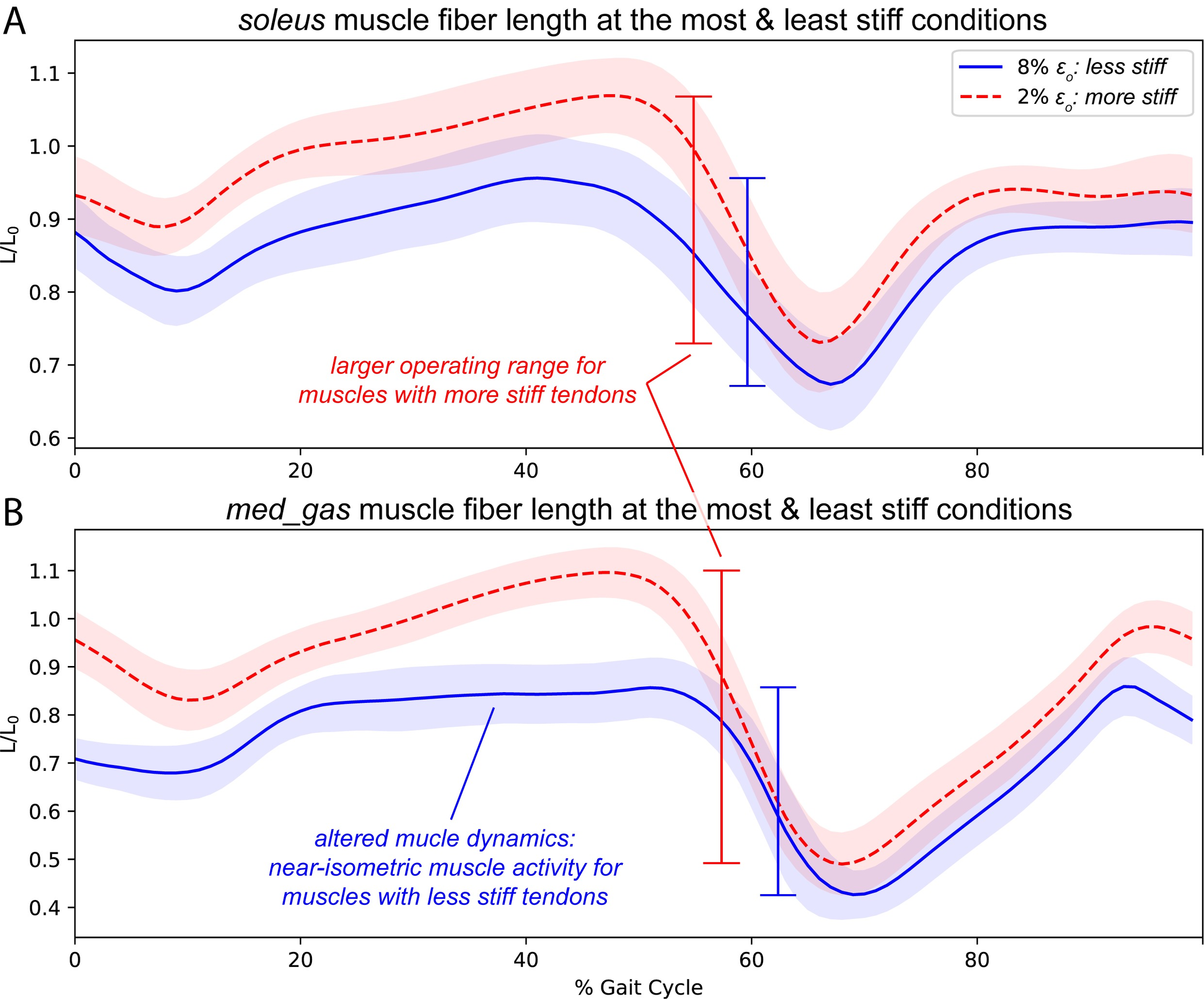

Supplement: S7 Fig — To show the effects of kT on muscle length more clearly, this figure focusses on the soleus (A) and med_gas (B) muscles for the Norm FP condition. Both subplots show the average relative muscle fiber length with shaded ±1 standard deviation across the gait cycle for the least stiff (8% εo, blue solid line) and most stiff (2% εo, red dashed line) conditions. We remind readers that kinematics (and thus MTU lengths) are constrained for all simulations. Comparing the two kT extrema, we see a clear reduction in operating range for the 8% εo condition across both muscles, implying that the more compliant tendon would elongate and recoil more in contribution to MTU length changes. Conversely, a stiffer tendon is unlikely to experience as much length change, requiring its respective muscle to undergo more shortening and lengthening. Indeed, in the least-stiff condition, the med_gas (B) acts nearly isometrically during mid-stance (20–50% gait cycle). (TIF) [file pone.0293331.s007.tif]
