# Supplementary material for: Simulations suggest walking with reduced propulsive force would not mitigate the energetic consequences of lower tendon stiffness
Source: PLoS One. 2023 Oct 26;18(10):e0293331. doi: 10.1371/journal.pone.0293331 (PMC10602298; doi:10.1371/journal.pone.0293331)
Supplement: S1 Table — We display how these individual muscles respond to changes in FP, kT, and interaction by reporting the ANOVA main effect (p-value) and effect size (ɳp2). Bolded muscle names indicate the top 12 consumers of metabolic cost, highlighted in Figs 4 & 5. (DOCX) [file pone.0293331.s008.docx]

**Supplementary Table 1:** We show the average activation level for all modeled muscles, averaged across the gait cycle. We display how these individual muscles respond to changes in F_P_, k_T_, and interaction by reporting the ANOVA main effect (p-value) and effect size (*ɳ_p_^2^*). Bolded muscle names indicate the top 12 consumers of metabolic cost, highlighted in Figures 4 & 5.

| **Rank** | **Muscle** | **Average Activation Level at Default** | **F_P_** | | **k_T_** | | **Interaction** | |
| --- | --- | --- | --- | --- | --- | --- | --- | --- |
|  |  |  | *p* | *ɳ_p_^2^* | *p* | *ɳ_p_^2^* | *p* | *ɳ_p_^2^* |
| - | MEAN | 32% | **<0.001** | **0.402** | 0.704 | 0.047 | 0.718 | 0.065 |
| 1 | **glut_med** | 67% | **0.002** | **0.309** | 0.325 | 0.098 | 0.990 | 0.031 |
| 2 | glut_min | 32% | **0.000** | **0.402** | 0.704 | 0.047 | 0.718 | 0.065 |
| 3 | **glut_max** | 31% | **0.008** | **0.262** | **<0.001** | **0.413** | 0.395 | 0.088 |
| 4 | **tib_ant** | 26% | 0.120 | 0.150 | **<0.001** | **0.729** | **<0.001** | **0.208** |
| 5 | **med_gas** | 21% | 0.375 | 0.090 | **<0.001** | **0.436** | **0.048** | **0.135** |
| 6 | **rect_fem** | 20% | 0.244 | 0.114 | **0.005** | **0.285** | 0.565 | 0.076 |
| 7 | **psoas** | 20% | 0.568 | 0.063 | **<0.001** | **0.477** | 0.089 | 0.123 |
| 8 | peri | 20% | 0.180 | 0.130 | **<0.001** | **0.449** | 0.444 | 0.084 |
| 9 | **iliacus** | 19% | 0.397 | 0.087 | **<0.001** | **0.413** | 0.334 | 0.093 |
| 10 | **bifemsh** | 18% | 0.648 | 0.054 | **<0.001** | **0.424** | 0.067 | 0.129 |
| 11 | ext_dig | 17% | 0.164 | 0.135 | **<0.001** | **0.692** | **0.043** | **0.137** |
| 12 | **soleus** | 15% | **0.030** | **0.212** | **0.001** | **0.338** | 0.398 | 0.088 |
| 13 | lat_gas | 14% | 0.323 | 0.099 | **0.001** | **0.328** | **0.025** | **0.146** |
| 14 | tfl | 14% | 0.488 | 0.073 | **<0.001** | **0.470** | 0.199 | 0.106 |
| 15 | sar | 14% | 0.663 | 0.052 | 0.273 | 0.108 | 0.956 | 0.041 |
| 16 | ercspn | 13% | 0.385 | 0.088 | 0.123 | 0.149 | 0.540 | 0.078 |
| 17 | **semimem** | 12% | 0.137 | 0.144 | **<0.001** | **0.599** | 0.551 | 0.077 |
| 18 | add_mag | 11% | **0.001** | **0.356** | **0.013** | **0.245** | 0.269 | 0.099 |
| 19 | quad_fem | 11% | 0.560 | 0.064 | 0.759 | 0.041 | 0.532 | 0.078 |
| 20 | intobl | 10% | 0.105 | 0.156 | 0.051 | 0.189 | 0.937 | 0.045 |
| 21 | **vas_lat** | 8% | **0.011** | **0.251** | **0.001** | **0.332** | **0.001** | **0.194** |
| 22 | ext_hal | 8% | 0.218 | 0.120 | **<0.001** | **0.590** | 0.511 | 0.080 |
| 23 | gem | 8% | 0.686 | 0.049 | **<0.001** | **0.461** | 0.879 | 0.052 |
| 24 | **bifemlh** | 8% | **0.028** | **0.215** | **<0.001** | **0.377** | 0.200 | 0.106 |
| 25 | add_long | 7% | 0.348 | 0.094 | **0.003** | **0.298** | 0.903 | 0.049 |
| 26 | vas_int | 7% | **0.005** | **0.285** | **0.001** | **0.337** | **0.001** | **0.202** |
| 27 | extobl | 7% | **0.006** | **0.277** | **0.002** | **0.317** | 0.937 | 0.045 |
| 28 | vas_med | 7% | **0.007** | **0.268** | **0.002** | **0.315** | **<0.001** | **0.212** |
| 29 | per_tert | 6% | 0.217 | 0.120 | **<0.001** | **0.501** | 0.573 | 0.075 |
| 30 | tib_post | 5% | 0.065 | 0.179 | **<0.001** | **0.471** | 0.097 | 0.121 |
| 31 | semiten | 5% | 0.597 | 0.060 | **<0.001** | **0.509** | 0.252 | 0.100 |
| 32 | per_long | 5% | 0.095 | 0.161 | **0.001** | **0.350** | 0.132 | 0.115 |
| 33 | flex_hal | 4% | **0.006** | **0.272** | **<0.001** | **0.615** | **0.030** | **0.143** |
| 34 | add_brev | 4% | 0.472 | 0.076 | 0.372 | 0.090 | 0.463 | 0.083 |
| 35 | flex_dig | 4% | **0.015** | **0.241** | **<0.001** | **0.856** | **<0.001** | **0.237** |
| 36 | grac | 4% | 0.383 | 0.089 | **<0.001** | **0.383** | 0.221 | 0.104 |
| 37 | pect | 3% | 0.444 | 0.080 | 0.609 | 0.058 | 0.463 | 0.083 |
| 38 | per_brev | 3% | 0.199 | 0.125 | **0.015** | **0.241** | 0.644 | 0.071 |
