# Supplementary material for: Simulations suggest walking with reduced propulsive force would not mitigate the energetic consequences of lower tendon stiffness
Source: PLoS One. 2023 Oct 26;18(10):e0293331. doi: 10.1371/journal.pone.0293331 (PMC10602298; doi:10.1371/journal.pone.0293331)
Supplement: S2 Table — We display how these individual muscles respond to changes in FP, kT, and interaction by reporting the ANOVA main effect (p-value) and effect size (ɳp2). Bolded muscle names indicate the top 12 consumers of metabolic cost, highlighted in Figs 4 & 5. (DOCX) [file pone.0293331.s009.docx]

**Supplementary Table 2:** We show the average normalized fiber length for all modeled muscles, averaged across the gait cycle. We display how these individual muscles respond to changes in F_P_, k_T_, and interaction by reporting the ANOVA main effect (p-value) and effect size (*ɳ_p_^2^*). Bolded muscle names indicate the top 12 consumers of metabolic cost, highlighted in Figures 4 & 5.

| **Rank** | **Muscle** | **Average Relative Fiber Length at Default** | **Fp** | | **kT** | | **Interaction** | |
| --- | --- | --- | --- | --- | --- | --- | --- | --- |
|  |  |  | *p* | *ɳ_p_^2^* | *p* | *ɳ_p_^2^* | *p* | *ɳ_p_^2^* |
| 1 | tib_post | 1.086 | **<0.001** | **0.381** | **<0.001** | **0.787** | **<0.001** | **0.356** |
| 2 | semiten | 1.061 | 0.121 | 0.150 | **<0.001** | **0.403** | 0.420 | 0.086 |
| 3 | ercspn | **1.043** | **0.018** | **0.233** | **<0.001** | **0.379** | 1.000 | 0.008 |
| 4 | extobl | 1.023 | **0.042** | **0.197** | **<0.001** | **0.481** | **0.025** | **0.146** |
| 5 | per_long | 1.004 | **0.044** | **0.196** | **<0.001** | **0.698** | **<0.001** | **0.299** |
| 6 | glut_min | 0.991 | 0.493 | 0.073 | **<0.001** | **0.402** | 0.251 | 0.100 |
| 7 | per_tert | 0.987 | **<0.001** | **0.625** | **<0.001** | **0.593** | **0.003** | **0.180** |
| 8 | per_brev | 0.982 | 0.265 | 0.110 | **<0.001** | **0.471** | **0.004** | **0.176** |
| 9 | gem | 0.971 | **0.033** | **0.208** | **<0.001** | **0.546** | 0.143 | 0.113 |
| 10 | tfl | 0.970 | 0.198 | 0.125 | **<0.001** | **0.686** | **0.003** | **0.179** |
| 11 | **glut_med** | **0.950** | **0.009** | **0.259** | **<0.001** | **0.498** | **0.029** | **0.143** |
| 12 | **iliacus** | **0.936** | **0.001** | **0.340** | **<0.001** | **0.579** | 0.499 | 0.080 |
| 13 | **bifemlh** | **0.931** | **0.133** | **0.145** | **<0.001** | **0.542** | 0.125 | 0.116 |
| 14 | **soleus** | **0.928** | **<0.001** | **0.609** | **<0.001** | **0.771** | **<0.001** | **0.295** |
| 15 | flex_dig | 0.916 | **<0.001** | **0.476** | **<0.001** | **0.919** | **<0.001** | **0.610** |
| 16 | **bifemsh** | **0.915** | 0.211 | 0.122 | **<0.001** | **0.566** | **0.003** | **0.177** |
| 17 | flex_hal | 0.906 | **<0.001** | **0.486** | **<0.001** | **0.791** | **<0.001** | **0.371** |
| 18 | ext_hal | 0.902 | **<0.001** | **0.605** | **<0.001** | **0.720** | **<0.001** | **0.254** |
| 19 | intobl | **0.899** | 0.095 | 0.161 | **<0.001** | **0.466** | 0.164 | 0.110 |
| 20 | ext_dig | **0.891** | **<0.001** | **0.603** | **<0.001** | **0.812** | **<0.001** | **0.342** |
| 21 | grac | 0.884 | 0.908 | 0.022 | **0.001** | **0.346** | 0.426 | 0.086 |
| 22 | add_brev | 0.880 | 0.947 | 0.016 | **0.007** | **0.270** | 0.633 | 0.071 |
| 23 | quad_fem | 0.879 | 0.011 | 0.253 | **0.015** | **0.241** | 0.985 | 0.034 |
| 24 | **rect_fem** | **0.872** | 0.060 | 0.182 | **<0.001** | **0.668** | 0.055 | 0.132 |
| 25 | **psoas** | **0.872** | **0.001** | **0.359** | **<0.001** | **0.627** | **0.048** | **0.135** |
| 26 | lat_gas | **0.859** | **<0.001** | **0.444** | **<0.001** | **0.754** | **<0.001** | **0.304** |
| 27 | sar | **0.845** | 0.191 | 0.127 | **<0.001** | **0.457** | 0.962 | 0.040 |
| 28 | **med_gas** | **0.840** | **0.000** | **0.450** | **<0.001** | **0.867** | **<0.001** | **0.419** |
| 29 | peri | 0.825 | 0.347 | 0.095 | **<0.001** | **0.857** | **<0.001** | **0.389** |
| 30 | add_mag | 0.780 | 0.593 | 0.060 | **0.003** | **0.298** | 0.885 | 0.051 |
| 31 | **semimem** | **0.772** | 0.200 | 0.125 | **<0.001** | **0.726** | **0.006** | **0.169** |
| 32 | **tib_ant** | **0.769** | **<0.001** | **0.624** | **<0.001** | **0.822** | **<0.001** | **0.354** |
| 33 | vas_int | **0.757** | 0.139 | 0.143 | **<0.001** | **0.479** | 0.479 | 0.082 |
| 34 | **vas_lat** | **0.747** | 0.107 | 0.156 | **<0.001** | **0.521** | 0.301 | 0.096 |
| 35 | vas_med | **0.745** | 0.142 | 0.142 | **<0.001** | **0.457** | 0.569 | 0.076 |
| 36 | add_long | **0.714** | **0.009** | **0.261** | **<0.001** | **0.477** | 0.693 | 0.067 |
| 37 | **glut_max** | **0.686** | **0.004** | **0.288** | **<0.001** | **0.384** | 0.778 | 0.061 |
| 38 | pect | 0.645 | **0.001** | **0.342** | **0.001** | **0.334** | 0.986 | 0.033 |
