# Supplementary material for: Simulations suggest walking with reduced propulsive force would not mitigate the energetic consequences of lower tendon stiffness
Source: PLoS One. 2023 Oct 26;18(10):e0293331. doi: 10.1371/journal.pone.0293331 (PMC10602298; doi:10.1371/journal.pone.0293331)
Supplement: Attachment — Submitted filename: Simulations suggest walking with reduced propulsive force would not mitigate the energetic consequences of lower tendon stiffness.docx [file pone.0293331.s010.docx]

**General questions:**

The study engendered a sense of interest as a result of its well-crafted composition and effectively presented findings. The entirety of the methodological approach and the resultant outcomes are deemed satisfactory. Nonetheless, I have compiled a series of general and specific comments aimed at enhancing the manuscript's overall quality. The primary objective of the investigation revolves around ascertaining potential correlations between diminished horizontal ground reaction force exerted on the foot and decreased tendon stiffness to decrease metabolic cost of walking. Through the examination of this hypothesis, the authors meticulously scrutinized young, healthy individuals and endeavored to extrapolate their findings to the elderly population by employing musculoskeletal simulations.

The study's purpose and hypothesis were designed to answer a very specific question: "**Could walking with a reduced F_p_ mitigate the metabolic penalty we pay for reduced k_t_?**".

However, the method and results section includes extra information unrelated to the core research question. Notably, the mention of increased **F_p_** beyond normal levels (i.e., +20% and +40%) lacks prior discussion in the introduction and abruptly emerges in the subsequent method and results section without appropriate contextualization for the reader. Consequently, I strongly urge the author to either provide a rationale and hypothesis for investigating the purpose of the study in relation to the increased **F_p_** or consider removing the increased **F_p_** from the study.

All participants initially determined their preferred walking speed during overground walking, which was subsequently employed as their default speed on the treadmill (referred to as **F_p_** normal). Subsequently, the biofeedback system was utilized to modify the **F_p_** level, adjusting it to ±20% and ±40% of the **F_p_** normal value. While it is true that the walking speed remained constant (corresponding to the individuals' preferred walking speed), it is important to investigate whether the kinematic parameters, such as ankle, knee, and hip joint angles, retained the same range of motion.

It is widely recognized that even minor ankle joint rotations can substantially influence maximal tendon strain (Arampatzis et al., 2008). Moreover, significant differences in the joint range of motion for the lower extremities have been observed between young and elderly individuals (Fukuchi & Duarte, 2008). Thus, it is imperative to provide further clarification regarding this apparent discrepancy and its implications on the study's findings.

The authors answered the main question of the study (i.e., Could walking with a reduced F_p_ mitigate the metabolic penalty we pay for reduced k_t_?) to a solid "**No**". I think it can increase the impact and strength of the manuscript if the authors indicate which muscle-tendon (or group of muscle-tendon such as triceps-surae and Achilles tendon) can mitigate the increased metabolic cost of the muscle-tendon (muscle-tendon group) due to reduced k_t_ by diminished F_p_. For instance, the GM muscle in -20 % seems to be the answer.

**Specific questions:**

Line 130: I find it challenging to comprehend the rationale behind the utilization of the metabolic energy expenditure system in your study, considering that neither its role as a validation tool for the model (as previously conducted) nor its output data are intended to be reported.

Line 142: I would appreciate a more detailed explanation regarding the origin or basis of the 2.0% value for the default maximum tendon strain in the **gait 2392** model, considering that the default value for all muscles in the model is fixed at 3.3%.

Figure 3: As a suggestion: Put the SPM significance bars on the bottom of the figure. In this way, the significant difference in the percentage of the gait cycle is more readable (it can be applied to other figures too). Or put the gait cycle ticks (5 ticks) on top of the SPM bar (I would prefer this one).

Figure 4: As a suggestion: I would like to see how the GL muscle reacts to the deviated **F_p_** from normal and reduced **K_t_** of the Achilles tendon (I expect to see something similar to GM). I know you have prioritized the muscle based on their metabolic expenditure, but the results of GL as one of the triceps surae muscle components can be interesting.

Arampatzis, A., De Monte, G., & Karamanidis, K. (2008). Effect of joint rotation correction when measuring the elongation of the gastrocnemius medialis tendon and aponeurosis. *Journal of Electromyography and Kinesiology*, *18*(3), 503-508.

Fukuchi, R. K., & Duarte, M. (2008). Comparison of three-dimensional lower extremity running kinematics of young adult and elderly runners. *Journal of sports sciences*, *26*(13), 1447-1454.
